# The Bethesda System for Reporting Thyroid Cytopathology: A Cytohistological Study

**DOI:** 10.1155/2020/8095378

**Published:** 2020-04-16

**Authors:** Bakiarathana Anand, Anita Ramdas, Marie Moses Ambroise, Nirmal P. Kumar

**Affiliations:** ^1^Department of Pathology, Pondicherry Institute of Medical Sciences, Ganapathichettikulam, Kalapet, Puducherry 605014, India; ^2^Department of General Surgery, Pondicherry Institute of Medical Sciences, Ganapathichettikulam, Kalapet, Puducherry 605014, India

## Abstract

**Introduction:**

The Bethesda System for Reporting Thyroid Cytopathology (TBSRTC) is a significant step to standardize the reporting of thyroid fine needle aspiration (FNA). It has high predictive value, reproducibility, and improved clinical significance.

**Aim:**

The study was aimed to evaluate the diagnostic utility and reproducibility of “TBSRTC” at our institute. *Methods and Material*. The study included 646 thyroid FNAs which were reviewed by three pathologists and classified according to TBSRTC. Cytohistological correlation was done for 100 cases with surgical follow-up and the sensitivity, specificity, positive predictive value, negative predictive value, diagnostic accuracy, and risk of malignancy (ROM) were calculated. The interobserver variation among three pathologists was also assessed.

**Results:**

The distribution of cases in various TBSRTC categories is as follows: I—nondiagnostic 13.8%, II—benign 75.9%, III—atypia of undetermined significance (AUS)/follicular lesion of undetermined significance (FLUS) 1.2%, IV—follicular neoplasm (FN)/suspicious for follicular neoplasm (SFN) 3.7%, V—suspicious for malignancy (SM) 2.6%, and VI—malignant 2.8%. The sensitivity, specificity, positive predictive value, negative predictive value, and diagnostic accuracy are 72.4%, 94.3%, 84%, 89.2%, and 87.9%, respectively. The ROM of various TBSRTC categories were II—8.5%; III—66.7%; IV—63.6%; and V and VI—100%. Cohen's Weighted Kappa score was 0.99 which indicates almost perfect agreement among the three pathologists.

**Conclusions:**

Our study substantiates greater reproducibility among pathologists using TBSRTC to arrive at a precise diagnosis with an added advantage of predicting the risk of malignancy which enables the clinician to plan for follow-up or surgery and also the extent of surgery.

## 1. Introduction

Thyroid nodules are a common clinical problem. It is important to differentiate benign from malignant nodules. Fine needle aspiration (FNA) is utilized as a preoperative diagnostic technique which is safe, simple, and cost effective for triaging patients with thyroid nodules [1].

Proper communication among pathologists, clinicians, radiologists, and surgeons along with cytohistological correlation is essential for reporting of thyroid FNA. Hence, consistent diagnostic terminology is vital.

To achieve standardization of diagnostic terminology, morphologic criteria, and risk of malignancy for reporting of thyroid FNA, in 2007, the National Cancer Institute (NCI) organized the NCI Thyroid Fine Needle Aspiration State of the Science Conference which proposed a 6-tier system and named it The Bethesda System for Reporting Thyroid Cytopathology (TBSRTC). The categories and their risk of malignancy for I—nondiagnostic, II—benign, III—atypia of undetermined significance (AUS)/follicular lesion of undetermined significance (FLUS), IV—follicular neoplasm (FN)/suspicious for follicular neoplasm (SFN), V—suspicious for malignancy (SM), and VI—malignant were 1–4%, 0–3%, 5–15%, 15–30%, 60–75%, and 97–99%, respectively [2].

The study aimed to evaluate the diagnostic utility and reproducibility of “The Bethesda System for Reporting Thyroid Cytopathology” at our institute.

## 2. Materials and Methods

All thyroid FNA smears and thyroidectomy specimens received from January 2013 to June 2018 in the Department of Pathology, at our institute, were included in the study after obtaining approval from the Institute Ethics Committee. The FNA smears were reviewed and categorized according to the Bethesda system. Cytohistological correlation was done for cases with surgical follow-up.

Statistical analysis was performed using R software version 3.5.1 (R Core Team) and Microsoft Office Excel 2007. Mean, median, and Standard Deviation (SD) were calculated for continuous variables like age. Categorical variables were expressed as frequencies and percentages. ANOVA test was used to calculate the *p* value. A *p* value <0.05 was considered statistically significant.

The diagnostic values (sensitivity, specificity, positive predictive value, negative predictive value, and accuracy) and risk of malignancy for FNAs using the Bethesda system were calculated for cases with surgical follow-up. FNA smears interpreted as nondiagnostic were excluded. True negative cases were defined as nodules with benign FNA cytology and surgical pathology. Follicular neoplasm/suspicious for follicular neoplasm, suspicious for malignancy, and malignant cases confirmed to be malignant upon final histology were considered true positive. Nodules with cytological results of FN/SFN or suspicious for malignancy or malignant diagnosed as benign on surgical excision were interpreted as false positive. False negative samples included cases with benign cytology that were found to be malignant upon histopathology.

Cross tabulation and Cohen's Weighted Kappa (*κ*) were applied to evaluate the concordance among the three observers. The Kappa coefficient was interpreted as follows: 0–0.2 indicates poor agreement, 0.3–0.4 indicates fair agreement, 0.5–0.6 indicates moderate agreement, 0.7–0.8 indicates strong agreement, and >0.8 indicates almost perfect agreement.

## 3. Results

The study included 646 patients with complaints of thyroid swelling evaluated by FNA. The age group of the patients ranged from 7 to 85 years with a mean of 41.78 years. The male: female ratio was 1 : 6.3.

### 3.1. Distribution of Cases according to the Bethesda System

Out of 646 cases, 75.9% were benign of which 34.7% was nodular goitre. Scant cellularity contributed with 7.8% of the nondiagnostic category. The distributions of AUS/FLUS (III) and FN/SFN (IV) were 1.2% and 3.7%, respectively. Category-V constituted 2.4% cases suspicious for papillary carcinoma. Papillary carcinoma (2%) was the most common malignancy in category-VI (Table 1).

### 3.2. Cytohistological Correlation with Assessment of Risk of Malignancy and Risk of Neoplasm

Cytohistological correlation was done for 100 patients with surgical follow-up. On histopathology, 71 cases were confirmed to be benign of which the most common was nodular goitre. Out of 100 cases, 29 were malignant. Papillary carcinoma (17%) was the most common malignancy followed by follicular carcinoma (6%) (Table 2).

Risk of malignancy was assessed for 100 cases with surgical follow-up. Out of 100 cases, one was excluded since it was reported as nondiagnostic on cytology. To calculate the risk of neoplasm the surgical resections were divided into three groups: benign nonneoplastic lesions, benign neoplasms, and malignant lesions (Table 2).

### 3.3. Determination of Diagnostic Values

The total of 99 cases was divided into two groups. One group comprised of Bethesda categories II and III for which surgery is not recommended due to low malignancy risk and the other group consisted of Bethesda categories IV, V, and VI for which surgery is recommended due to high malignancy risk. The sensitivity, specificity, positive predictive value, negative predictive value, and diagnostic accuracy hence obtained are 72.4%, 94.3%, 84%, 89.2%, and 87.9%, respectively (Table 3).

### 3.4. Interobserver Agreement

Cross tabulation and Cohen's Weighted Kappa (*κ*) were applied to evaluate the concordance among the three observers. Cohen's Weighted Kappa score was 0.99 which indicates almost perfect agreement among the three pathologists.

## 4. Discussion

The goal of thyroid FNA is to successfully differentiate benign from malignant lesions and to triage patients requiring surgery. The six-tired Bethesda system provides standardized nomenclature for reporting thyroid FNA smears which enables better communication and understanding between clinicians and pathologists. The advantage of this systematic approach is that each of the six Bethesda categories has implied risk of malignancy which helps the clinicians to plan appropriate therapy necessary for the patient [3].

Nondiagnostic (ND) thyroid FNA result remains a major constraint in arriving at a definitive diagnosis and is the most common cause of false negative reports [4]. It is difficult to assess the risk of malignancy for the ND category because only a small subset of ND nodules undergoes resection. Hence there is disparity in the malignancy rate among various studies which ranges from 0% to 63.2% [5, 6].

Gunes et al. stated that the clinical expertise of the person performing the FNA, ultrasound guidance, and rapid on-site evaluation for specimen adequacy were not uniform between studies which contributes to the wide range of malignancy rate. All these determinants make the comparison between studies cumbersome and should be taken into consideration while labelling a specimen as nondiagnostic and assessing the risk of malignancy [4]. Some of the studies stated that the operator experience and the number of passes made during FNA correlate with the nondiagnostic result [7, 8].

In our study, the nondiagnostic yield was 13.8% which was high when compared to TBSRTC consensus. Sampling error and technical quality due to the above-mentioned reasons and strict adherence to the adequacy criteria explain the high rate of ND smears.

Mondal et al. and Nandedkar et al. found high incidence of category II lesions since the patients directly visit a tertiary care center for primary diagnosis without any referral which was also the case in our study [6, 9].

The incidence of benign lesions in our study was 75.9% when compared to studies done in USA ranging from 64% to 66% which can be attributed to the regional variation in the incidence of thyroid disorders and where majority of patients come only on a referral basis and hence are not exactly representative of the general population [10, 11].

The implied risk of malignancy for category II is 0% to 3% with the recommended management being clinical follow-up of patients [2]. Although surgery is not recommended for category II lesions, the patients in our study were operated mainly for cosmetic purpose and pressure symptoms.

The indeterminate category, AUS/FLUS, has led to confusion due to inconsistent usage amongst pathologists of various institutions. This category should be used as a last resort in reporting with the expectation of 7% or less cases to receive this diagnosis as proposed by TBSRTC. Layfield et al. reported a variation of 2.5–28.6% among individual pathologists and 3.3–14.9% among three academic institutions [12].

There were less number of cases (1.2%) diagnosed under the category AUS/FLUS in our study which was due to rigid adherence to the diagnostic criteria and the pathologists endeavor to avoid ambiguity and keep the use of AUS/FLUS to a minimum which was in similarity to a study by Nandedkar et al. which had 0.8% of cases in category III out of 606 FNA's [9]. Jo et al. and Yassa et al. have reported 3.4% and 4% lesions as AUS/FLUS, respectively [13, 14].

Mondal et al. reported a lower percentage (1%) of AUS/FLUS cases which was a result of performing ultrasound guided FNA in small and heterogeneous nodules with suspicious features on palpation and radiological evaluation, so that the aspirate can be obtained from the exact site of lesion which is a routine practice even at our institute [6].

The actual risk of malignancy of category III is difficult to determine, since confirmatory diagnosis is only available in a subset of patients selected for surgery who have suspicious clinical or USG features. The patients are also subjected to selection bias which overestimates the prevalence of malignancy [15].

The risk of malignancy of AUS/FLUS cases was 69% in a study done by Park et al. which was higher when compared to our study and TBSRTC guidelines. This was because patients with high index of clinical suspicion for malignancy undergo surgery without a repeat FNA. Patients tend to be more concerned about false positive results than false negative results, which might have pressurized cytopathologists to underdiagnose cases to avoid making false positive diagnosis [16].

**Figure 1:**
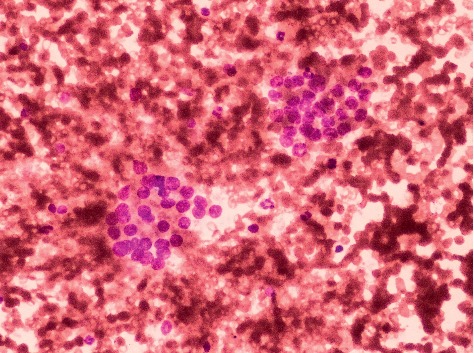
Atypia of undetermined significance (Bethesda category III). Smear shows clotting artefact with crowding of follicular cells hindering the interpretation (MGG stain ×400).

Our study was held in a teaching hospital, where FNAs were performed by different persons with varied level of experience during their training period. This factor could have resulted in hemodilution and artefactual changes during smear preparation which might have contributed to a higher ROM in category III (Figure 1). Repeat FNAs of such cases along with clinicoradiological correlation could have decreased the proportion of cases reported in this category as well as the ROM.

**Figure 2:**
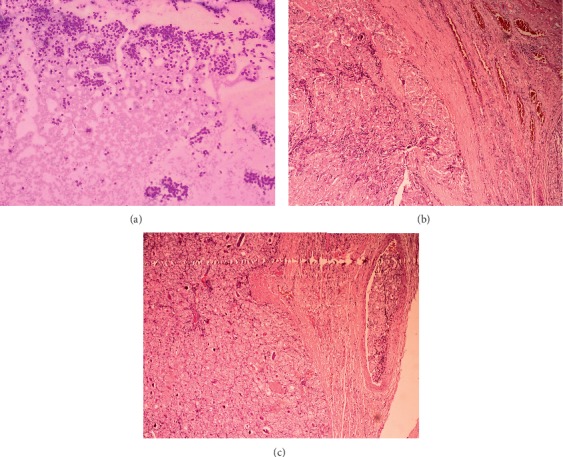
Follicular neoplasm/suspicious for follicular neoplasm (Bethesda category IV). (a) Highly cellular smear with cells arranged predominantly in microfollicular pattern (MGG ×100). Histopathology of the same showed follicular carcinoma with capsular invasion (b) and vascular invasion (c) (H&E ×100).

Based on cytology it is difficult to distinguish follicular carcinoma from follicular adenoma [2, 12] (Figure 2). Melo-Uribe et al. correlated the results of thyroid FNA reported using the TBSRTC with histopathology, from three different hospitals in Columbia. There was significant variation in the malignancy risk of category IV which measured 56.3% in oncology centers and 23.5% in nononcology centers which was attributed to the selection bias of the patients requiring surgery [17].

The high ROM in categories III and IV in our study when compared to other studies may be due to the following reasons. Firstly, it is due to the heterogeneity of the indeterminate categories III and IV which are subject to variation in interpretation across institutions [3]. Secondly, it is because of variations in number of patients with surgical follow-up and also the selection bias of patients requiring surgery.

Our study had 2.4% cases suspicious for papillary thyroid carcinoma (PTC) which was similar to the lower range of rate of suspicious for PTC in the following study [15]. The ROM of category V in a study by Williams et al. was less when compared to our study which may be due to variation in cohort characteristics and underdiagnoses of lesions leading to hemithyroidectomy rather than total thyroidectomy [18].

**Figure 3:**
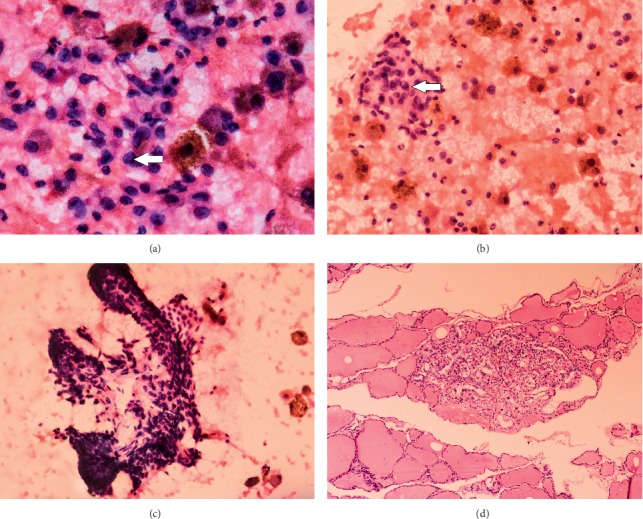
Suspicious for papillary carcinoma (Bethesda category V). (a) One of the follicular cells show nuclear groove (arrow) (H&E ×400). (b) Intranuclear cytoplasmic inclusion (arrow) seen in occasional follicular cell (H&E ×400). (c) Smear shows focal papillaroid structure (H&E ×400). (d) Histopathology of the same showed papillary microcarcinoma (H&E ×100).

The ROM in a study by Partyka et al. was in good correlation with our study in categories V and VI which was 100% each after inclusion of papillary microcarcinoma [19] (Figure 3). Our study was able to accurately predict the ROM for suspicious for malignancy and malignant nodules due to the practice of correlating cytologic features with clinical, biochemical, and USG findings while reporting (Table 2).

The risk of neoplasm (RON) gives an overall estimate of predicting both benign and malignant lesions. Our study had nil risk of neoplasm in the nondiagnostic category (Table 2). This was due to repeat FNA of cases with high index of clinical and ultrasound features suspicious for malignancy.

The RON of category II was similar to the study done by Wu et al. (Table 4) [20]. This was due to false negative reporting of 2 papillary microcarcinoma, 1 Hurthle cell carcinoma, and 1 follicular carcinoma as benign. Two cases of conventional papillary carcinomas were misdiagnosed as benign due to sampling error (Table 2). Follicular carcinoma and Hurthle cell carcinoma are difficult to diagnose on FNA and need to be confirmed by histopathology. Papillary microcarcinoma is a lesion that measures 1 cm or less which can be easily missed on FNA unless the aspirator hits the target.

Our study was able to accurately predict the RON of categories III, V, and VI when compared to the study done by Wu et al. which could be attributed to the routine practice of correlating cytology with clinical, biochemical, and radiological features at our institute (Table 4) [20].

The FN/SFN category had RON of 81.8% which was high compared to the study by Wu et al. This was due to classification of two cases of nodular goitre as category IV lesion (Table 2). Another possible reason could be the variation in sample size and less number of cases with surgical follow-up in our study (Table 4) [20].

Mehra and Verma in their study found that the method of statistical analysis can alter the results of diagnostic values. If suspicious lesions are considered positive, the sensitivity increases while the specificity decreases. If suspicious lesions are excluded, then the sensitivity decreases and the false negative rates increase. In their study diagnostic values were calculated by either excluding FN/SFN or including it with either benign or malignant diagnosis to highlight the effect on diagnostic values [21].

Shi et al. suggested that eliminating the diagnosis of category III substantially decreases the sensitivity of thyroid FNAs (the sensitivity for detecting PTC dropped from 100% to 27%) and increases both false positive and false negative rates. The authors concluded that AUS/FLUS category should not be eliminated but recommended using it minimally [22].

The findings from our study indicate that the calculation of sensitivity, specificity, positive predictive value, negative predictive value, and diagnostic accuracy of thyroid FNAs according to the Bethesda system are less reliable because of the arbitrary nature of cases classified under categories III (AUS/FLUS) and IV (FN/SFN) (Table 3).

The main purpose of TBSRTC was to eliminate the ambiguity and to follow uniformity in the reporting of thyroid FNAs thereby enabling ease of communication among pathologists and clinician and to plan appropriate treatment for the patients [2].  Table 5 shows comparison of interobserver reproducibility of our study with that of other studies [23–25].

Our study differed from a study done by Padmanabhan et al. which assessed the interobserver reproducibility in reporting AUS/FLUS category among seven cytopathologists which revealed fair agreement (Fleiss kappa score 0.23) and recommended review of AUS/FLUS cases for more definite categorization [25]. We observed a trend that the less the number of observers (2-3), the more the chance of interobserver agreement (Table 5).

## 5. Conclusion

Thyroid FNA smears reported using the Bethesda system helped in achieving more precise cytological diagnosis. Our study substantiates greater reproducibility among pathologists using TBSRTC for reporting thyroid FNA. The Bethesda system has an added advantage of predicting the risk of malignancy which enables the clinician to plan for follow-up or surgery and also the extent of surgery.

## Figures and Tables

**Table 1:** Distribution of cases according to the Bethesda system.

Bethesda category	Bethesda category percentage (%)	FNA diagnosis	No. of cases (total = 646)	Percentage (%)
I—nondiagnostic (89)	13.8	Cyst fluid	6	0.9
		Scant cellularity	50	7.8
		Obscuring blood	33	5.1
II—benign (490)	75.9	Nodular goitre	224	34.7
		Adenomatoid nodule	37	5.7
		Colloid nodule	70	10.8
		Grave's disease	3	0.5
		Lymphocytic (Hashimoto) thyroiditis	156	24.2
III—AUS/FLUS (8)	1.2	AUS/FLUS	8	1.2
IV—FN/SFN (24)	3.7	FN/SFN	24	3.7
V—suspicious for malignancy (17)	2.6	Suspicious for papillary carcinoma	16	2.4
		Suspicious for medullary carcinoma	1	0.2
VI—malignant (18)	2.8	Papillary carcinoma	13	2.0
		Medullary carcinoma	3	0.4
		Poorly differentiated carcinoma	1	0.2
		Undifferentiated carcinoma	1	0.2

**Table 2:** Cytohistological correlation with assessment of risk of malignancy and risk of neoplasm.

Bethesda category	No. of cases (total = 646)	Cases that underwent surgery (total = 100)	Histopathology diagnosis			Risk of neoplasm (%)	Risk of malignancy including papillary microcarcinoma (%)	Risk of malignancy excluding papillary microcarcinoma (%)
			Benign nonneoplastic	Benign neoplastic	Malignant lesion			
I—non diagnostic	89 (13.8%)	1	Colloid nodule (1)	0	0	0	0	0
II—benign	490 (75.9%)	71	Nodular goitre (42)	Follicular adenoma (4)	Follicular carcinoma (1)	14.1	8.5	5.6
			Adenomatoid hyperplasia (10)		Papillary carcinoma (2)			
			Colloid nodule (5)		Papillary microcarcinoma (2)			
			Lymphocytic/Hashimoto thyroiditis (4)		Hurthle cell carcinoma (1)			
III—AUS/FLUS	8 (1.2%)	3		Follicular adenoma (1)	Follicular carcinoma (1)	100	66.7	66.7
					Papillary carcinoma (1)			
IV—FN/SFN	24 (3.7%)	11	Nodular goitre (2)	Follicular adenoma (1)	Follicular carcinoma (4)	81.8	63.6	63.6
				Hurthle cell adenoma (1)	Papillary carcinoma (2)			
					Medullary carcinoma (1)			
V—suspicious for malignancy	17 (2.6%)	7			Papillary carcinoma (5)	100	100	85.7
					Papillary microcarcinoma (1)			
					Medullary carcinoma (1)			
VI—malignant	18 (2.8%)	7			Papillary carcinoma (7)	100	100	100

**Table 3:** Determination of diagnostic values.

Test	HPE malignant	HPE benign	Total
FNA Bethesda categories IV, V, VI	21	4	25
FNA Bethesda categories II, III	8	66	74
Total	29	70	99

**Table 4:** Comparison of risk of neoplasm of our study with another study by Wu et al. [20].

Bethesda category	Risk of neoplasm of our study (%) (*n* = 100/646)	Risk of neoplasm in a study by Wu et al. (%) (*n* = 221/1382)
I—nondiagnostic	0	24
II—benign	14.1	14
III—AUS/FLUS	100	44
IV—FN/SFN	81.8	67
V—SFM	100	77
VI—malignant	100	100

**Table 5:** Comparison of interobserver reproducibility of among various studies.

Study	No. of observers	Interobserver agreement
Awasthi et al. [23]	2	Good (Cohen's kappa score 0.613)
Padmanabhan et al. [25]	7	Fair (Fleiss kappa score 0.23)
Pathak et al. [24]	3	Strong (Fleiss kappa score 0.6561)
Our study	3	Almost perfect (Cohen's kappa score 0.99)

## Data Availability

The raw data used to support the findings of this study have not been made available because of patient's confidentiality and privacy rules.

## Additional Points

The yield of nondiagnostic aspirate was high due to the varied experience level of the persons who performed the thyroid FNA. Repeat USG guided FNA would have reduced the number of nondiagnostic aspirates but it was feasible only for patients with high index of clinical and radiological features suspicious of malignancy.
